# Applying the Integrated Practice Unit Concept to a Modified Virtual Ward Model of Care for Patients at Highest Risk of Readmission: A Randomized Controlled Trial

**DOI:** 10.1371/journal.pone.0168757

**Published:** 2017-01-03

**Authors:** Lian Leng Low, Shu Yun Tan, Matthew Joo Ming Ng, Wei Yi Tay, Lee Beng Ng, Kanchana Balasubramaniam, Rachel Marie Towle, Kheng Hock Lee

**Affiliations:** 1 Department of Family Medicine & Continuing Care, Singapore General Hospital, Singapore; 2 Family Medicine, Duke-NUS Medical School, Singapore; 3 Health Services Research, Medicine Academic Clinical Program, Singapore General Hospital, Singapore; Public Library of Science, FRANCE

## Abstract

**Background:**

Emerging evidence from the virtual ward care model showed that multidisciplinary case management are inadequate to reduce readmissions or death for high risk patients. There is consensus that interventions should encompass both pre-hospital discharge and post-discharge transitional care to be effective. Integrated practice units (IPU) had been proposed as an approach of restructuring the organization and work processes of multidisciplinary teams to achieve value in healthcare. Our primary objective is to evaluate if the novel application of the IPU concept to organize a modified virtual ward model incorporating pre-hospital discharge transitional care can reduce readmissions of patients at highest risk for readmission.

**Methods:**

We conducted an open label, assessor blinded randomized controlled trial on patients with one or more unscheduled readmissions in the prior 90 days and LACE score ≥ 10. 840 patients were randomized in 1:1 ratio and blocks of 6 to the intervention program (n = 420) or control (n = 420). Allocation concealment was effected via an off-site telephone service maintained by a hospital administrator. Intervention patients received discharge planning, medication reconciliation, coaching on self-management of chronic diseases using standardized action plans and an individualized care plan complete with written discharge instructions, appointments schedule, medication changes and the contact information of the outpatient VW nurse before discharge. At discharge, care is handed over to the outpatient VW team. Patients were closely monitored in the VW for three months that included a telephone review within 72 hours of discharge, home assessment, regular telephone reviews to identify early complications and early review clinics for patients who destabilize. The VW meet daily to discuss new patients and review care plans for patients. Control patients received standard hospital care that included a standardized patient copy of the hospital discharge summary listing their medical diagnoses and medications; and follow up is arranged with a primary care provider or specialist as considered necessary. The primary outcome was the unplanned readmission rate to any hospital within 30 days of discharge. Secondary outcomes included the unplanned readmission rate, emergency department (ED) attendance rate to any hospital and the probability without readmission or death up to 180 days of discharge. Length of stay and mortality rate at 90-day were compared between the two groups. Outcome data were objectively retrieved from the hospital and National Electronic Health Records by a blinded outcome assessor.

**Findings:**

All patients’ outcomes were included in an intention-to-treat analysis. The characteristics of both study groups were similar. Patients in the intervention group had a significant reduction in the number of 30-day readmissions, IRR 0.67 (95% CI, 0.52 to 0.86, p = 0.001) and the number of 30-day emergency department attendances, IRR 0.60 (95% CI, 0.46 to 0.79, p<0.001) compared to those receiving standard hospital care. The effectiveness was sustained at 90 and 180 days. The intervention group utilized 1164 fewer hospital bed days at 90-day post discharge. No adverse events were reported.

**Conclusion:**

Applying the integrated practice unit concept to the virtual ward program resulted in reduced readmissions in patients who are at highest risk of readmission.

## Introduction

Integrated care is widely accepted as the solution that will enable health systems to cope with the escalating demands for hospital resources as the population ages rapidly and health care needs become increasingly complex. Care integration is expected to achieve better health outcomes and reduce cost by improving system efficiency [1–3]. However, evaluations of integrated care programs are often lacking in rigor and the few effective programs have targeted patients at average risk of readmission [4–6]. The virtual ward intervention was first described and implemented in the United Kingdom in 2006 as an improvement over case management [7]. The virtual ward model consists of two fundamental components: (1) using a predictive tool to identify patients who are at high risk of future unplanned readmission in that virtual ward’s catchment area; and (2) using the systems, staffing and daily routines of a hospital ward to offer a period of intensive, multi-disciplinary case management to these high-risk patients to reduce their risk of unplanned readmissions. Patients remain in the community during their admission to a virtual ward. The virtual ward model was well received at its launch, receiving multiple awards for innovation in providing post-discharge care [8]. Despite the attractiveness of the concept, a formal evaluation commissioned by the Department of Health in 2013 for the participating sites found no evidence of reduction in unplanned readmissions when compared to matched controls [9]. Similarly, two large randomized clinical trials conducted independently in Canada and Singapore on a post-discharge virtual ward model were unable to reduce readmissions or death for high risk patients [10, 11].

Transitional care concepts have been recommended to reduce readmissions and its value well described by Kripalani et al [12]. Emerging evidence suggest that interventions may be more effective when they are in the form of multi-component bundles [12–14] and started early in the care cycle [4, 12]. Key components include discharge planning, medication safety, dedicated transitional care personnel, coordination among team members, patient empowerment for self-management and follow-up care [12]. Despite the increasing awareness of the importance of integrating care across the care cycle, healthcare workers on the ground continue to encounter barriers including ineffective communication within the multidisciplinary team in the hospital and with primary care providers in the community [15]. In our modified virtual ward intervention, we sought to improve its effectiveness by incorporating transitional care interventions early in the care cycle before the patient is discharged from the hospital.

Integrated practice units (IPU) had been proposed as a way of restructuring multidisciplinary teams to increase value in healthcare [16]. The IPU requires structure and processes to be organized along a conceptual framework that is designed to achieve common outcomes. In an IPU, personnel work together as a team towards a common goal: maximising the patient’s overall outcomes as efficiently as possible. IPU members are co-located to facilitate communication, collaboration and efficiency for patients. Initially, integrated practice units emerge as multi-disciplinary teams defined around medical conditions or closely related medical conditions that involve similar skills, facilities and care delivery approaches. Condition-based IPUs proliferated rapidly across many areas of acute and chronic care, from organ transplantation to shoulder care and mental health conditions such as eating disorders. More recently, Porter et al who originated this concept had proposed the extended application to segments of patient population with common care needs in primary care [17]. There is reason to believe that the IPU concept can work well for patients who are at high risk for readmissions and require transitional care. While there are anecdotal reports of good outcome with the IPU approach to multidisciplinary care for breast cancer and low back pain, to our knowledge IPUs have not been organized to provide care integration initiatives for patients at high risk of unplanned readmissions with complex chronic conditions.

The primary aim of our study is to evaluate the effectiveness of our integrated practice unit and modified virtual ward model that incorporates pre-hospital discharge transitional care concepts in reducing acute hospital utilization of patients at highest risk of readmission.

## Methods

### Study Setting and Trial Methodology

Singapore is a multi-ethnic Asian population of 5.6 M people. Its population is one of the most rapidly ageing in Asia with an increasing chronic disease burden [18]. Care is fragmented and episodic and the lack of integration between the well-developed tertiary hospitals and less-developed primary and community care sectors is well acknowledged. 30-day readmission rates in the elderly are high at 19.0% [19] and only slightly lower than in the United States [20]. Healthcare expenditure is expected to triple from S$4 billion in 2011 to S$12 billion in 2020 [21] and driven mainly by inpatient cost. Concerns about long term sustainability of current trends is driving a concerted search for new models of care delivery which reduces the dependence on high cost hospitals care. There is great interest in developing transitional care programs to improve patient outcomes and reduce wasteful utilization of expensive hospital resources. Taking a population health approach, Singapore created six regional health systems (RHSs) in 2011, each being responsible to integrate care for a specific geographic region.

Singapore General Hospital (SGH) is the largest tertiary hospital with 1597 beds, accounting for about 20% of the total public acute hospital beds in Singapore. SGH is the flagship hospital of the SingHealth regional health system. It has 36 specialist departments, including family medicine. SGH is wholly owned by the Ministry of Health Singapore. With a workforce numbering above 10,000, SGH admits over 1 million patients every year at its wards, emergency department and outpatient specialist clinics [22]. Since 2006, the hospital embarked on developing new models of care integration that will reduce care fragmentation and improve care continuity. As part of the hospital’s effort to innovate and deliver better transitional care models, we incorporated the IPU framework (Table 1) to a virtual ward care model to achieve closer administrative and physical integration of our multidisciplinary team that is responsible for care transitions.

**Table 1:** 10 Attributes of Integrated Practice Units (IPUs) as applied to our modified Virtual Ward Model.

Attribute of IPU	Characteristic of our modified Virtual Ward Model
Organized around a medical condition or population segments with similar care needs	Patients who are at high risk of unplanned re-admissions with a common need for coordinated integrated care interventions to reduce readmission risk.
Multidisciplinary team with dedicated time to the team	Physicians, nurses, and medical social workers spend dedicated time on our team. Therapists and pharmacists are assigned on an ad-hoc basis to ensure cost-effective use of manpower.
Common organizational unit	Clinical and administrative staff are organized to and funded by the hospital unit responsible for integrated care.
Team takes responsibility for the full cycle of care	The cycle of care is extended beyond the traditional virtual ward care model to include the post-acute phase of hospitalization where intensive discharge planning is provided by members of the IPU.
Education, engagement and follow up of patients are part of the team’s responsibility	The modified virtual ward model is responsible for the education, engagement and follow up of patients.
Single administrative and scheduling structure	Administrative support staff members are integrated with the clinical staff into a single administrative and scheduling structure. All staff are involved in all aspects of planning and work process development and implementation.
Co-located in dedicated facilities	The team is co-located in a common operations centre in the hospital.
Physician team captain or clinical care manager oversees care process	The team is led by a family physician who works closely with integrated care nurses who oversees each virtual ward.
Common outcome measures	The team share common outcome measures to reduce hospital utilization for their patients. Work load and resources are tracked and shared with the team at regular administrative meetings.
Regular formal and informal meetings	Multidisciplinary virtual ward meetings are held daily. Formal monthly meetings are held for briefings. Informal ad-hoc meetings and retreats are organized to improve processes and overcome administrative barriers to care.

We conducted an open label, outcome assessor blinded, parallel-group randomized trial comparing the modified virtual ward model of care with usual care from October 2013 to December 2014 at the Singapore General Hospital.

### Inclusion Criteria and Exclusion Criteria

Patients were eligible if they were aged 21 years or older, being admitted to participating medical wards (general internal medicine, endocrinology, respiratory medicine, renal medicine, gastroenterology, neurology), at high risk of readmission (as determined by LACE score ≥ 10 [length of stay, acuity of the admission, charlson comorbidity score, and emergency department visits in the previous 6 months] score ≥10 [22] and at least ≥1 admission in the previous 90 days). The LACE index (Length of stay, Acuity of admission, Charlson comorbidity index, Emergency department visits in past 6 months) was a readmission predictive score derived in Ontario, Canada with a score ranging from 0 to 19. Patients with a score of ≥ 10 were found to have a five times higher risk of 30-day readmission in Singapore [22]. When a LACE score ≥ 10 was combined with one or admissions in the previous 90 days, patients had a 30% risk of an unplanned 30-day readmission [11]. Eligible case finding and risk stratification using the LACE index and prior admissions in the previous 90 days is performed electronically by the hospital’s enterprise analytics platform eHIntS (Electronic Health Intelligence System) on a daily basis. Potentially eligible patients were screened in the wards for further exclusion criteria by a research coordinator.

Patients were excluded if they were critically ill at the time of screening: hemodynamically unstable as defined by requiring inotropes; acute respiratory support with ventilators or FiO2 more than 50%; acute dialysis support or care in the high dependency or intensive care unit. Other exclusion criteria are requiring surgical intervention at the time of screening; discharged or deceased before they could be screened for eligibility; non-resident; no telephone contact or local home address for post-discharge surveillance; impaired decision making capacity without a legal surrogate decision maker to provide consent; or if they did not wish to participate. Finally, patients who are admitted from a long term care facility were excluded as it was not possible to deliver our post-discharge interventions to this group of patients. Patients who met both inclusion and exclusion criteria were approached for written informed consent. For patients unable to provide consent, it was obtained from a legal substitute decision maker.

### Randomization

Consented patients were randomized in a 1:1 ratio and blocks of six using a computer-generated randomization list to either our modified virtual ward model of care or standard care. Allocation concealment was effected via an off-site telephone service maintained by a hospital administrator. The generation of the random allocation sequence and enrolment of patients was performed by a research coordinator. Given the nature of the intervention, it was not possible to blind patients or clinicians. However, the outcome assessor and statistician were blinded to the treatment assignment.

### Ethics Approval and Trial Registration

Research ethics approval was granted by the Institutional Review Board of Singapore Health Services. Our trial was not registered prospectively due to a genuine administrative error by our administrator (Clinicaltrials.gov, no NCT02351648) and it was never our intention to publish only if we had positive findings. We had also published our earlier trial which did not have a significant result [11]. We noticed the error late and immediately registered our trial upon discovery of our error. The ethics committee approved the study on 11^th^ October 2012. We recruited our first patient on the 24^th^ October 2012 and completed the follow up for our last patient on the 6^th^ January 2015. The authors confirm that all ongoing and related trials for this drug/intervention are registered.

### Trial Intervention, Control and Outcomes

#### Intervention

1. Integrated Practice Unit (IPU) Line-up and Roles and Responsibilities of team members: The intervention consisted of a multidisciplinary team of integrated care nurses, pharmacists, medical social workers organized into an IPU led by attending family physicians. The IPU comprised of an inpatient care team and an outpatient virtual ward (VW) team. The IPU is co-located in the same physical locality; share a common electronic patient record and a common mission to reduce avoidable readmissions (Table 1). An attending family physician, medical officer, a nurse case manager, and a part-time (0.1 Full Time Equivalent) pharmacist formed the inpatient team while the outpatient VW team comprised of an attending family physician and two nurse case managers. A medical social worker who spends 0.5 full time equivalent time in the team supports both the inpatient and outpatient teams in activating the appropriate community and social services that supports the patient in the home setting. Family physicians in SGH function as hospitalists providing general medical care to medical inpatients [23], including follow-up care of patients in the outpatient specialist clinic upon hospital discharge. The nurse case managers’ main function is to provide discharge planning and case management to patients who are admitted in the hospital or in the virtual wards. Patient education and activation are their part of their core activities. In addition they can provide short term nursing care when required. Pharmacists assist in medication reconciliation and advise on potentially harmful drug adverse events and interactions. The physiotherapist and occupational therapist assess and train patients and their caregivers in mobility and activities of daily living respectively. The speech therapist assess and manage swallowing and communication disorders including training to patients and their caregivers to minimize aspiration risk.

2. Pre-discharge Hospital phase: Upon assignment to the intervention group, patients had their hospital care transferred to the inpatient care team of the IPU. In addition to providing general medical care, the team systematically identified and addressed patient risk factors for readmission and focused on intensive discharge planning. Medications were reconciled and the appropriate community and social services necessary to support the patient’s transition to home were activated. A core component is patient education and coaching. The nurse case managers used standardized action plans for chronic diseases (examples include congestive cardiac failure, diabetes mellitus, asthma, chronic obstructive pulmonary disease, chronic kidney disease) for patient education. Other comorbid medical conditions including mental health conditions were managed holistically, including ensuring appropriate specialist follow up on discharge. Finally, an individualized care plan complete with written discharge instructions, patients’ appointments, medication changes and the contact information of the outpatient nurse case manager is provided to all patients on discharge.

3. Post-discharge Outpatient Phase: On the day of discharge, the inpatient nurse case manager introduces the patient to the outpatient nurse case manager and hands over care of the patient to the outpatient team. The outpatient nurse case manager follows up with a telephone call within 72 hours of discharge to assess the patient’s condition and ensure adherence to the prescribed care plans and successful activation of community services.

A home assessment is performed by the outpatient nurse case manager within one week of discharge. During the home assessment, the nurse case manager assessed the patient’s medical condition and health literacy, competency of the care-giver, availability of nursing and home care equipment, adequacy of social support, safety of the home environment and adherence to medication. The nurse case manager addresses any identified areas of deficiency, with help from the multidisciplinary team. Referrals were made to activate social and financial support services when needed. Each nurse was responsible for an average of 30 patients at any time during the program.

A multidisciplinary team meeting was conducted in the morning of every working day. New patients are brought up for discussion and care plans were reviewed. During the meeting, each nurse case manager will also report on the status of patients under her care. Patients with urgent problems are referred to the early review clinic. Patients who are doing well are reviewed less frequently at the team meetings. Throughout the intervention period, the nurse case managers are contactable during office hours. Scheduled calls to check in on the patients are made about once a week Patients were discharged at the end of the intervention period of three months to a primary care provider in the community with continuing specialist input as warranted.

#### Control

Patients in the control group received standard hospital care. On discharge, patients may be referred to primary care provider, specialists in the outpatient clinic and ambulatory community services as considered necessary by the medical team. Patients receive an abbreviated standardized patient copy of the hospital discharge summary listing their medical diagnoses and medications. For this study, there was no contact between patients in the control group and the study team throughout the 3-month interval. A scheduled telephone call was made at the end of 3 months when patients or their caregivers were assessed for various outcomes.

#### Outcomes

The primary outcome was the unplanned readmission rate to any hospital within 30 days of discharge. An unplanned readmission is defined as an emergency admission from the emergency department or outpatient specialist clinic. Secondary outcomes included the unplanned readmission rate to any hospital within 90 days, 180 days of discharge, as well as emergency department (ED) attendance rate within 30 days, 90 days, 180 days of discharge and the probability without readmission or death up to 180 days. Index admission and post-discharge mortality rate at 90-day; Length of stay and number of outpatient specialist clinic visits at 90-day and 180-day were compared between the two treatment groups. For patients who died during the index admission, the count for the primary and secondary outcomes would be zero. For patients who died during the 90 days follow up, the count for primary and secondary outcomes would censored at the time of death. In addition, we performed cost analysis on the costs of intervention and hospital services utilization in both intervention and control groups. The cost of intervention is computed from the costs of additional components in the intervention, absent in standard hospital care. These included the additional manpower for the post-discharge virtual ward phase and the additional nurse case manager, social worker and pharmacist support for the hospital phase. We did not include physician costs for the hospital phase in both the intervention and control groups as they are primarily employed by the hospital to care for inpatients. We assumed that the hospitalization costs fully accounted for the salaries of physicians in both intervention and control groups. We estimated the potential cost savings to the patient from the difference in hospital bed days, emergency department attendances saved against the additional costs of outpatient specialist clinic visits at 90 days post-discharge. Outpatient specialist clinic visit cost, Emergency department visit cost and average bed cost per patient day in 2013 were S$75, S$216 and S$1075 respectively [24, 25]. Outcome data were objectively retrieved from the hospital’s electronic data repository and the National Electronic Health Records (NEHR) by an outcome assessor blinded to the group assignment. The hospital’s electronic data repository and the NEHR captured all admissions and ED attendances to SingHealth hospitals and all hospitals in Singapore respectively, and is available for all patients including those who were uncontactable, or transferred to specialist discipline during index admission, or transferred to long term care facility.

### Sample Size

We hypothesized that our integrated practice unit and modified virtual ward model could reduce readmissions by approximately 25% for patients enrolled in the intervention group. We estimated the baseline readmission rate of eligible patients to be 0.4 per patient within 30 days of discharge. Power was calculated at 80%, level of significance at 5%, and attrition at 10%, resulting in a required a sample size of 420 in each group. The statistical analysis was performed on an intention-to-treat basis.

### Statistical Analysis

The baseline characteristics were presented as mean ± SD for continuous variables and frequency counts and percentages for categorical variables. A negative binomial regression model was used to obtain incidence rate ratios (IRRs) of readmission, ED visits and outpatient specialist clinic visits. A zero-inflated negative binomial regression model is used if there is a statistically significant excess-zero problem for any of the readmission, ED visit or outpatient specialist clinic visit outcomes. An exponential hurdle regression model was used to obtain the IRRs of length of stay within different pre-defined time periods. Kaplan-Meier method was used to study the survival distribution (based on time to readmission or death) of the intervention and control groups from the date of index discharge (day 1) to 180 days post-discharge. The hazard ratio on time to readmission or death is calculated using the Cox proportional hazard model. If patients did not experience the event (readmission or death) by the end of the follow-up period, they were censored at the end of the time period. All tests of significance used 95% level (p<0.05). All analyses were performed using STATA 14.0.

## Results

**Fig 1:**
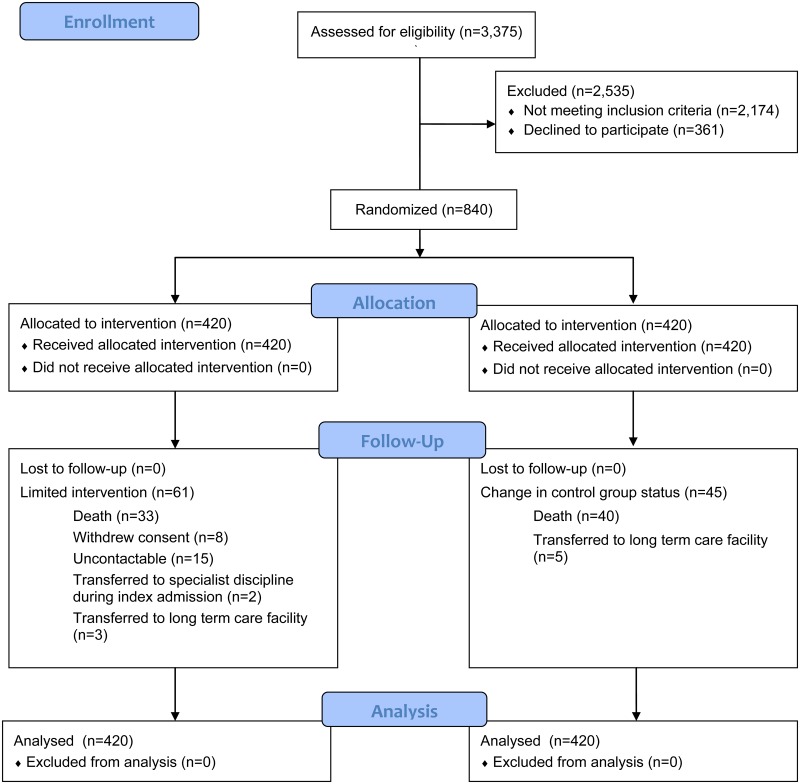
CONSORT Flow Diagram.

Between October 2013 and December 2014, 3376 patients met the inclusion criteria performed electronically by the hospital’s enterprise analytics platform eHIntS and were screened further in the wards for eligibility. Of the 1201 eligible patients, 840 consented to be randomized into 1 of the 2 groups (Fig 1). All patients’ outcomes were available from the hospital electronic data repository and the NEHR and included in an intention-to-treat analysis.

### Study Population

The characteristics of both study groups were similar at baseline (Table 2). The mean age (SD) of patients was 70.5 (13.5) and 70.3 (13.7) years in the intervention and control groups respectively. The majority of the patients were of Chinese race, married, had lower education status, stayed in subsidized ward classes and public housing flats. The average Charlson Comorbidity index, LACE index and hospital utilization in the preceding 90 days prior to index admission were similar in both study groups. Both groups were similar in terms of comorbidities such as heart failure, diabetes mellitus, and stroke except for chronic obstructive pulmonary disease which was more prevalent in the intervention group.

**Table 2:** Baseline Characteristics of Participants.

Baseline characteristics		Treatment Plan	
		Control (n = 420)	Intervention (n = 420)
Age mean (SD)		70.3 (13.7)	70.5 (13.5)
Male Gender n (%)		199 (47)	211 (50)
Ethnicity	Chinese	281 (67)	299 (71)
	Malay	70 (17)	54 (13)
	Indian	61 (14)	58 (14)
	Other	8 (2)	9 (2)
Marital status n (%)	Single	35 (8)	35 (9)
	Married	246 (60)	247 (61)
	Widowed	115 (28)	104 (26)
	Separated/Divorced	17 (4)	17 (4)
Need for a caregiver n (%)	No	9 (2)	12 (3)
	Yes	320 (76)	306 (75)
	Self-care	91 (22)	91 (22)
Admissions ward class† n (%)	A1	14 (3)	7 (2)
	B1	29 (7)	36 (8)
	B2	200 (48)	167 (40)
	C	171 (41)	201 (48)
	Others	6 (1)	8 (2)
House ownership n (%)	Owned	364 (87)	342 (83)
	Rented	39 (9)	51 (12)
	Others	15 (4)	20 (5)
Index admission LOS mean (SD)		7.54 (10.60)	6.92 (7.37)
Charlson Comorbidity score mean (SD)		3.27 (1.76)	3.29 (1.70)
LACE score mean (SD)		12.76 (2.15)	12.72 (2.20)
Congestive heart failure n (%)		123 (29)	127 (30)
Diabetes Mellitus n (%)		253 (60)	242 (58)
Cerebrovascular accident n (%)		83 (20)	85 (20)
Chronic obstructive pulmonary disease n (%)		14 (3)	28 (7)
Admission Specialty before modified virtual ward n (%)	General Internal Medicine	399 (95.0)	404 (96.2)
	Endocrine	2 (0.5)	1 (0.2)
	Gastroenterology	5 (1.2)	6 (1.4)
	Neurology	4 (1.0)	1 (0.2)
	Respiratory Medicine	10 (2.4)	8 (1.9)

^†^ In Singapore, admission ward classes are categorized according to different levels of government subsidies. Ward classes A, B1, B2 and C received a government subsidy of 0%, 20%, 65% and 80%, respectively.

LOS = Length of Stay

### Primary Outcome

As Table 3 shows, the number of 30-day readmissions in the intervention group was 33% lower than that in the control group. The mean hospital readmission per patient reduced from 0.38 in the control group to 0.25 in the intervention group.

**Table 3:** Analysis of Primary and Secondary Outcomes.

Outcome Variables	Intervention (mean, SD)*	Control (mean, SD)*	IRR^†^ (95% CI)	P value
Hospital Readmissions within 30 days	0.25 (0.54)	0.38 (0.63)	0.67 (0.52,0.86)	0.001
Hospital Readmissions within 90 days	0.67 (1.01)	0.90 (1.26)	0.74 (0.61–0.90)	0.001
Hospital Readmissions within 180 days	1.05 (1.45)	1.46 (1.96)	0.72 (0.60–0.86)	<0.001
ED visits within 30 days	0.26 (0.51)	0.43 (1.16)	0.60 (0.46–0.79)	<0.001
ED visits within 90 days	0.66 (1.02)	0.92 (1.64)	0.72 (0.58–0.89)	0.001
ED visits within 180 days	1.14 (1.64)	1.60 (2.34)	0.71 (0.60–0.85)	<0.001
			Marginal Mean^††^	P value
Hospital LOS within 30 days	1.98 (6.16) ^ⱡ^	3.10 (6.84) ^ⱡ^	-1.37 (-2.18, -0.56)	<0.001
Hospital LOS within 90 days	5.29 (10.86) ^ⱡ^	7.44 (13.42) ^ⱡ^	-2.42 (-4.07, -0.76)	0.004
Hospital LOS within 180 days	8.70 (16.96) ^ⱡ^	12.10 (20.88) ^ⱡ^	-3.60 (-6.08, -1.12)	0.004

* mean = visits/patient/month after index hospitalization

^†^ IRR = Incidence Rate Ratio, obtained from negative binomial regression unless otherwise stated.

^††^Marginal Mean: obtained from exponential hurdle regression

ED = Emergency Department

LOS = Length of Stay

^ⱡ^ = mean hospital bed days/patient/month after index hospitalization

### Secondary Outcomes

**Fig 2:**
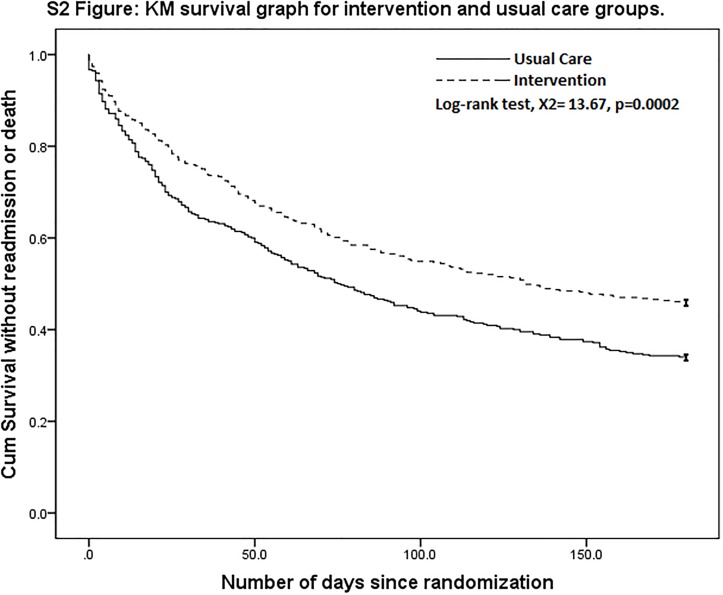
Kaplan-Meier Survival Curve on Probability without Readmission or Death.

This trend had by and large remained at both 90 days and 180 days post discharge with 26% and 28% reduction respectively in the number of readmissions as a result of the intervention. There was also a 28% reduction in mortality rate in the intervention group compared to the control group during the 180 days of follow up (HR 0.72; 95% CI, 0.61 to 0.86; *p* < 0.001) (Fig 2). Table 3 also shows a reduction in the number of ED attendances at the 30-day, 90-day and 180-day periods after discharge. The number of 30-day ED attendances for patients in the intervention group was 40% lower than that in the control group. Similar to the number of readmissions, the difference in the number of ED attendances between the intervention group and the control group became smaller over time but remained statistically significant at 28% and 29% respectively at 90 days and 180 days after discharge.

We also found a statistically significant reduction in inpatient length of stay within 30 days, 90 days and 180 days post discharge as a result of the intervention. At 30 days post discharge, patients in the intervention group had 1.37 fewer hospital bed days than patients in the control group. This difference increased to 2.42 days and 3.60 days respectively at 90 days and 180 days post discharge.

The intervention group appeared to have significantly more outpatient specialist clinic visits than the control group (see Table 4). However, after excluding visits to our early review clinic, we found the intervention group to have fewer outpatient specialist clinic visits compared to the control group, but the difference was not statistically significant.

**Table 4:** Analysis of Outpatient Specialist Clinic Visits.

Outpatient Specialist Clinic Visits	IRR^†^ (95% CI)	P value
Within 30 days	1.54 (1.31, 1.82)	<0.0001
Within 90 days	1.4 (1.24, 1.67)	<0.0001
Within 180 days	1.24 (1.07, 1.44)	0.005
**Sensitivity Analysis by excluding Early Review Clinic Visits**		
Within 30 days	0.87 (0.73, 1.04) ^††^	0.121
Within 90 days	0.89 (0.77, 1.03)	0.107
Within 180 days	0.90 (0.79, 1.03)	0.138

^†^ IRR = Incidence Rate Ratio, obtained from negative binomial regressions, unless stated otherwise.

^††^ IRRs were obtained from zero-inflated negative binomial regressions.

We found that the total costs of intervention and hospital services utilization in the intervention group was lower than the costs of hospital services utilization in the control group (Table 5). Compared to the control group, the intervention group had 1,164 fewer bed days from the index admission to 90 days post-discharge with an associated cost savings of S$1,250,655. In addition, there were 109 fewer ED attendances with associated cost savings of S$23,544. The additional cost of outpatient specialist clinic visits totalled S$33,675. The difference between study groups in total cost (savings from reduced hospital utilization and additional costs of outpatient specialist clinic visits was S$1,240,524 –an average of S$2,954 saved per patient who received the intervention. These savings have not included additional indirect cost savings as a result of lower hospital services utilization.

**Table 5:** Costs of intervention and hospital services utilization.

(A) Incremental cost of intervention over standard care						
Intervention costs^!^	Units		Unit cost per year (S$)	Cost for trial duration of 2 years (S$)		
Family Physician Associate Consultant	0.5*		236,000	236,000		
Family Physician Registrar	0.5*		107,800	107,800		
Nurse case manager	3*		58,000	348,000		
Medical Social Worker	0.5*		84,500	84,500		
Pharmacist	0.1*		99,380	19,876		
Administrator	0.5*		41,200	41,200		
Transport costs for nurse home assessment^ⱡ^	420		25	10,500		
Subtotal cost				847,876		
(B) Hospital services utilization costs						
	Intervention group			Control group		
Costs of hospital services utilization	Number	Unit cost (S$)	Total cost (S$)	Number	Unit cost (S$)	Total cost (S$)
Readmission bed days	5,128	1,075	5,512,815	6,292	1,075	6,763,470
Emergency Department visits	277	271	75,067	386	271	104,606
Outpatient specialist clinic visits	1,470	75	110,250	1,021	75	76,575
Total costs (A) + (B)			6,546,008	6,944,651		

^!^ = Based on actual average salary in Singapore General Hospital for 2013

* = Full Time Equivalent

^ⱡ^ = Total costs calculated based on one home assessment each for 420 intervention patients.

There were no significant differences between the 2 groups for deaths during the index admission (6 and 8 patients in the intervention and control group respectively; *p* = 0.590) and at 90 days post-discharge (33 and 40 patients in the intervention and control group respectively; *p* = 0.397).

## Discussion

Our study showed that the Integrated Practice Unit concept can be applied to the Virtual Ward model of care and reduces readmissions in patients who are at high risk of unplanned hospital readmissions. We found synergy when these two concepts were combined. While previous studies have focused on reengineering the discharge planning process [14] or improving case management in high risk patients [10], to our knowledge, our study is the first trial to evaluate an extended cycle of integrated care on patients at high risk of readmission. Patients at high risk of unplanned readmissions frequently suffer from complex co-morbidities which strain personal and community resources. The problem is by nature complex and requires complex and multimodal interventions. Our study adds to integrated care literature by demonstrating the clear need for high-intensity multimodal interventions to reduce readmissions in high risk patients.

There are several possible explanations for the success of our modified virtual ward model in reducing acute hospital utilization. First, we adopted a novel approach in organizing our inpatient care team and outpatient virtual ward team into an IPU that share common work space and processes created a positive culture of collaboration and teamwork. For example, the physical location of the virtual ward in the hospital allowed the outpatient VW team members to build rapport with the patients before discharge and receive timely handovers from the inpatient team members at the bedside. Second, whereas individual concepts might have limited effectiveness, combining compatible concepts such as the IPU and VW in care integration interventions may result in synergistic effects that exceed the sum of the parts in reducing hospital utilization of high risk patients. Third, the inpatient team members extended the cycle of care by taking over responsibility of patient care during the hospitalization phase of care for patients who were randomized to the intervention group. This allowed the IPU to address readmission risk factors in a systematic and concerted effort early in the care cycle [26] and the inclusion of pre-discharge transitional care concepts could have accounted for the difference between our results and Dhalla et al [10]. Low health literacy and medication adverse events are known risk factors for 30-day hospital utilization post discharge [27, 28]. The inpatient care team was able to address these areas through patient education, clear discharge instructions and medication reconciliation. Extending the cycle of care also provided additional lead time to activate necessary community and social care services to support patients upon discharge from hospital. Interestingly, the length of stay during the index admission was shorter in the intervention group when compared to the control group (Table 3). This may be due to the intensive and focused discharge planning which improved efficiency of care during the hospital phase. Fourth, the care at the Emergency Department is often constrained by the lack of ability to influence pre-attendance and post-attendance management of patient [29]; therefore the tendency to admit frail, medically complex patients is high. Our early review clinic provided an alternative to the Emergency Department for patients who developed acute issues or destabilization of their chronic conditions. Prompt intervention of exacerbations and decompensations in patients with chronic diseases by the early review clinic helped keep patients out of the hospital and reduce avoidable readmissions.

To date, there are limited clinical trials of care integration that had conclusively shown effectiveness in reducing readmissions [5, 6, 14]. It is important to note the significant differences between these trials and our study. Patients enrolled in these studies tend to be younger. In one such trial which focused on re-engineering discharge process [14], the median age of the subjects were 20 years younger than our study cohort. The subjects also had lower risk of unplanned re-admission. The average 30-day re-admission rate was 0.207 readmissions per patient in the control arm of this study. The average 30-day re-admission rate of patients in the control arm of our study was almost two times higher at 0.38 readmissions per patient. Similarly other successful studies also had subjects with lower risk of re-admission. The 30-day readmission rate of two such studies were lower at 11.3% and 14% respectively [5, 6].

The age, Charlson comorbidity score and mean LACE score in our study were most similar to the study population enrolled in Dhalla’s virtual ward intervention in Canada [10] and Lee’s transitional care intervention for patients at highest risk of readmission [11]. On the basis of the markedly different outcomes between the studies, it is reasonable to conclude that multicomponent, complex interventions starting before hospital discharge and extending across the care cycle are required to reduce readmissions substantially in high risk patients. Burke et al identified ten domains of intervention (Complete Communication of Information, Availability, Timeliness, Clarity, and Organization of Information, Medication Safety, Educating Patients to Promote Self-Management, Monitoring and Managing Symptoms after Discharge, Enlisting Help of Social and Community Supports, Advanced Care Planning, Coordinating Care Among Team Members, Discharge Planning, Follow-Up with Outpatient Providers) to be associated with reduction in readmissions and proposed the Ideal Transitions in Care framework based on these 10 domains[30]. Prior intervention studies addressed an average of 3.5 out of 10 domains. In contrast, 8 out of the 10 domains were core interventions in our study.

Singapore’s healthcare system is highly regarded in the world, coming in first among 51 countries ranked for efficiency of the healthcare system. The ranking was based on factors including life expectancy, the cost of health care as a percentage of gross domestic product and per capita health expenditure. For such good outcomes, Singapore’s expenditure on healthcare is among the lowest for developed countries. In 2014, the government health expenditure was a mere 1.9% of GDP [31]. The high level of efficiency had been attributed to Singapore’s approach towards healthcare financing. Singapore offers universal healthcare coverage but adheres to the philosophy of individual responsibility where citizens are expected to make substantial co-payment on top of government subsidies. This co-payment amount in turn may be reduced further by 3 major financing schemes. These are Medisave, MediShield Life and Medifund. Medisave is a mandatory health savings account whereby workers contribute part of their monthly salaries to a personal Medisave account. The savings can be withdrawn to pay the hospital bills of the account holder and immediate family members. Medishield Life is a basic health insurance plan which helps to pay for large hospital bills and selected costly outpatient treatments, such as dialysis and chemotherapy for cancer. The premiums are subsidized by the government and can be paid for through the patients’ own Medisave account. The affordability of the premiums is assured by the Government. Medifund is a safety net that is designed as a funding of last resort for patients who are needy and unable to pay despite optimal use of the other financial support schemes [32]. Despite the apparent high value that is achieved by the healthcare system, there are serious concerns that this is not sustainable in the future. Singapore has one of the world’s most rapidly aging population, due to a combination of increased life expectancy and falling fertility rates. The aging population is expected lead to an increase in healthcare expenditure largely due to increased utilization of hospital services. Total government health expenditure per capita rose from 2,310 USD in 2012 to 2,752 USD in 2014 [33]. This is expected to rise further to 3,232 USD in 2020 [21]. The reasons for this high rate of increase may be attributed to: a rapidly aging population with increasing chronic disease burden utilizing more hospital services; increased infrastructure and manpower spending to meet the healthcare needs of the aging population. Whilst increasing capacity to meet demand is inevitable, improving efficiency through reducing avoidable hospitalization and timely transition to more cost effective providers in the community is seen as a key strategy in sustaining the healthcare system into the future. Our program is the first in our country and in the region to convincingly show the effectiveness of transitional care program in terms of reduction of hospital utilization. The overall cost savings in our program suggest that our care model can be sustainable. Bundled payments that adequately remunerate health systems to provide the full cycle of transitional care should be encouraged to sustain these programs.

### Lessons Learnt

Health systems around the world are implementing various care integration and transitional care programs. The common objective is to reduce hospital utilization and develop a more sustainable healthcare system. Our program was based on adaptations of concepts and care models tried previously. Many such earlier projects had limited or no success, including our own earlier project which did not reduce hospital utilization significantly. Nevertheless, from ours and the experience of others, there are important lessons to be learnt for health systems designing similar interventions in future.

Firstly, concepts and models of care must be adapted or modified to local settings and guided by outcome measures. In our example, we modified the virtual ward model to include a pre-discharge hospital phase after our earlier trial [11] that followed the original virtual ward model failed to show improvements in hospital utilization. The IPU concept was originally developed to improve care for specific diseases. We applied it to our transitional care program by targeting patients at risk of re-admission. Interestingly, Porter et al who first described this concept subsequently used it in primary care to target segments of population with similar care needs.

Secondly, health systems implementing transitional care programs should consider extending the cycle of care to address risk factors at multiple points of the care continuum. In our trial, the addition of a pre-discharge hospital phase in our modified virtual ward model improved the effectiveness of the original model which focused mainly on post-discharge outpatient care integration.

Thirdly, transitional care is complex and requires a variety of skill sets in different care phases and settings. The need for multidisciplinary care is widely accepted. However having multidisciplinary care by itself is not enough. Transitional care works by re-integrating fragmented care as the patient enters different phases of the care cycle. The IPU is a framework that was conceptualized to improve complex care by multidisciplinary teams. Our study showed that it is an effective organizing principle for multidisciplinary teams that provide transitional care. Using this concept we organized our inpatient care team and the virtual ward team into a single IPU with good results.

### Strengths and Limitations

Our study had an important strength. We evaluated our modified virtual ward model of care using a randomized controlled trial design, which is the most rigorous design to minimize selection bias. This is critical for evaluation of complex interventions which is susceptible to bias from unknown confounding.

Our study also has several limitations. Firstly, our intervention was targeted at patients at highest risk of readmission and conducted at a single centre, therefore our findings may not be generalizable to all health systems without additional studies to confirm our findings. It is likely that a learning curve is required to replicate the complex nature of our interventions and the effectiveness may vary depending on level of staff competency and the organizational culture. Secondly, the success of our IPU model should have considered other short and long term health outcome measures such as health related quality of life and quality indicators of chronic diseases, in addition to reducing acute hospital utilization. In the short follow up period of 90 days in our trial, we had chosen to focus our study objectives on the effectiveness of delivering a modified virtual ward model of care to high-risk patients in reducing readmission risk.

### Practical Implications and Future Directions

Our study had shown that having multiple components and combining strategies in care integration produces synergistic effects. The constant search for single ingredients or a “magic bullet” in care integration may be a self-defeating mental model. For complex interventions, constant evaluation by comparison of outcome with controls is essential to provide feedback to continuously improve the working model and ensure that good outcomes are achieved. Although generalizability and reproducibility may be limited, valuable lessons can be learned through careful study and adaptation of useful elements to local organizations or health systems.

Future studies examining the extension of care model to include primary care should be considered. Decades of experience prove that a primary health care approach is the most efficient, fair and cost-effective way to organize a health system [31] and will be required to sustain the improvements beyond the transition period after discharge.

## Conclusion

Applying the integrated practice unit concept to the modified virtual ward program that incorporated pre-hospital discharge transitional care interventions reduced hospitalization in high risk patients by one third. Combining compatible concepts in care integration may produce synergistic results and more studies to confirm this finding would be useful. Care integration projects should adapt elements of promising new concepts to local settings and evaluate outcome using controlled studies.

## Supporting Information

S1 Text CONSORT checklist. (DOC)

S2 Text Study Protocol. (DOCX)

S1 Dataset Study Dataset. (XLSX)
