# Supplementary material for: Applying the Integrated Practice Unit Concept to a Modified Virtual Ward Model of Care for Patients at Highest Risk of Readmission: A Randomized Controlled Trial
Source: PLoS One. 2017 Jan 3;12(1):e0168757. doi: 10.1371/journal.pone.0168757 (PMC5207403; doi:10.1371/journal.pone.0168757)
Supplement: S2 Text — (DOCX) [file pone.0168757.s002.docx]

**
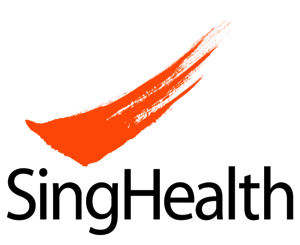
**

**STUDY PROTOCOL**

| **PROTOCOL TITLE:**  A Randomised Controlled Trial of a Transitional Care Model in Singapore | |
| --- | --- |
|  | |
|  | |
| **PROTOCOL VERSION:**  SGH_OIC_ICCS Version 1.0  **PROTOCOL DATE:**  1^ST^ September 2012 |  |
|  |  |
|  | |
| **PRINCIPAL INVESTIGATOR:** | |
| Associate Professor Lee Kheng Hock, Head & Senior Consultant, Department of Family Medicine and Continuing Care, Singapore General Hospital | |
|  | |
|  | |
|  | |
| **CO-INVESTIGATORS:** | |
|  | |
| Dr Tan Shu Yun, Consultant, Department of Family Medicine and Continuing Care, Singapore General Hospital | |
| Dr Low Lian Leng, Registrar, Department of Family Medicine and Continuing Care, Singapore General Hospital  Dr Tay Wei Yi, Registrar, Department of Family Medicine and Continuing Care, Singapore General Hospital  Dr Matthew Ng Joo Ming, Consultant, Department of Family Medicine and Continuing Care, Singapore General Hospital  Dr Ng Lee Beng, Associate Consultant, Department of Family Medicine and Continuing Care, Singapore General Hospital | |
|  | |
|  | |
|  | |
|  | |
|  | |

Table of Contents

1. BACKGROUND AND RATIONALE 3

2. HYPOTHESIS AND OBJECTIVES 4

3. EXPECTED RISKS AND BENEFITS 4

4. STUDY POPULATION 4

4.1. List the number and nature of subjects to be enrolled. 4

4.2. Criteria for Recruitment and Recruitment Process 5

4.3. Inclusion Criteria 5

4.4. Exclusion Criteria 5

5. STUDY DESIGN AND PROCEDURES/METHODOLOGY 5

6. DATA ANALYSIS 7

6.1. Data Entry and Storage 7

7. SAMPLE SIZE AND STATISTICAL METHODS 7

7.1. Determination of Sample Size 7

7.2. Statistical and Analytical Plans 8

8. DIRECT ACCESS TO SOURCE DATA/DOCUMENTS 8

9. ETHICAL CONSIDERATIONS 8

9.1. Informed Consent 8

9.2. Confidentiality of Data and Participant Records 8

10. FUNDING and INSURANCE 9

**11. APPENDIX 1 …………………………………………………………………………………… 10**

| **BACKGROUND AND RATIONALE** |  |
| --- | --- |
| Healthcare systems around the world are struggling to meet the increasing demand for hospital resources. Ageing populations and the increasing prevalence of chronic diseases and co-morbidities exacerbate this rising demand [1]. Singapore’s population is one of the most rapidly ageing in Asia with an increasing chronic disease burden [2, 3]. Care is fragmented and episodic and the lack of integration between the well-developed tertiary hospitals and less-developed primary and community care sectors is well acknowledged. There are concerns over the long term sustainability of a hospital centric care model; therefore there has been great interest to develop integrated care programs that provide more cost-effective care to high risk patients with multi-comorbidities and reducing the dependence on hospital resources.  The Virtual Ward model was first developed in Croydon, United Kingdom. While there are anecdotal reports of its effectiveness, the program has not been formally evaluated. Our hospital performed a randomized controlled trial using the virtual ward care model for high risk patients with a LACE score of 10 or more [4] in 2011, but did not find a significant reduction in readmissions and emergency department [ED] visits. It is possible that the virtual model of care is simply too weak an intervention to reduce readmissions for high risk patients with complex care needs. Emerging evidence also suggest interventions may be more effective when they are started early in the admission [5] and in the form of multi-component bundles [5, 6] instead of single interventions.  While there is face validity to these conceptual frameworks, delivering pre and post-discharge care in a coordinated manner has practical challenges in operations and logistics. The lack of horizontal integration between healthcare providers is well acknowledged as an impediment to achieving good outcomes in care integration initiatives. Integrated Practice Units (IPUs) have been proposed by Michael Porter as a way to organize healthcare teams [7]. With buy-in from the hospital management and relevant stakeholders, we propose to utilize the IPU concept to organize our inpatient clinical team and outpatient virtual ward team to deliver pre-discharge and post-discharge interventions in a coordinated manner in the Singapore General Hospital. The IPU would be led by a senior family physician, physically co-located, share a similar administration structure under an Office of Integrated Care, a common patient clinical record and be responsible for shared process and outcome indicators.  In this study, our objective is to study the effectiveness of an IPU in providing holistic care which starts during inpatient admission and continue this with seamless outpatient support from our virtual ward intervention. The primary outcome of interest would be the 30-day readmission rate.  References:  1. 1. National Institute on Aging, National Institutes of Health, U.S. Department of Health and Human Services. Global health and ageing report: The World Health Organization; 2011. Available from: http://www.who.int/ageing/publications/global_health.pdf.  2. Khalik S: Bed Crunch Time Again at Some Public Hospitals. The Straits Time [Singapore] 2011. http://www.healthxchange.com.sg/News/Pages/Bed-crunchtime- again-at-some-public-hospitals.aspx.  3. Khalik S: Public Hospital ‘borrowing’ Ward Space. The Straits Time [Singapore]  4. van Walraven C, Dhalla IA, Bell C, Etchells E, Stiell IG, Zarnke K, Austin PC, Forster AJ. Derivation and validation of an index to predict early death or unplanned readmission after discharge from hospital to the community. *CMAJ* 2010, 182(6):551-557.  5. Hansen LO, Young RS, Hinami K, Leung A, Williams MV. Interventions to Reduce 30-Day Rehospitalization: A Systematic Review. Ann Intern Med. 2011;155(8):520-8.  6. Jack BW, Chetty VK, Anthony D, Greenwald JL, Sanchez GM, Johnson AE et al. A reengineered hospital discharge program to decrease rehospitalization: a randomized trial. Ann Intern Med. 2009;150(3):178-87.  7. Porter ME, Teisberg EO. Redefining health care: Creating value-based competition on results. Boston, Massachusetts: Harvard Business School Press; 2006. |  |
| **HYPOTHESIS AND OBJECTIVES** |  |
| The main aim of our study is to find out if our integrated care model can reduce the rate of unscheduled readmission and emergency department visits of high risk patients in the Singapore General Hospital.  Based on our previous study, we hypothesize that our intervention could reduce 30-day readmission rate by approximately 25% for patients enrolled in the intervention group in order to be clinically significant.  Other secondary outcomes include:   1. Unplanned readmission rate to any hospital within 90 days, 180 days of discharge, 2. Emergency department (ED) attendance rate within 30 days, 90 days, 180 days of discharge 3. Probability without readmission or death up to 180 days 4. Index admission and post-discharge mortality rate at 90-day 5. Cumulative length of hospital stay at 90 days 6. Number of outpatient specialist clinic visits at 90-day and 180-day |  |
| **EXPECTED RISKS AND BENEFITS** |  |
| There is no higher than expected risk to study participants. |  |
| **STUDY POPULATION** |  |
| List the number and nature of subjects to be enrolled. |  |
| Patients who are eligible for the study would be all adults patients admitted to participating medical wards (general internal medicine, endocrinology, respiratory medicine, renal medicine, gastroenterology, neurology) in Singapore General Hospital, who has a LACE score of ≥10 and 1 or more unscheduled admission in the past 90 days.  Based on our sample size calculation, we will need to randomize a total of 840 patients in a 1:1 ratio to the intervention and control groups. |  |
| Criteria for Recruitment and Recruitment Process |  |
| The research coordinator will first enquire if the subjects are interested and willing to participate in the study. For eligible and consented patients, the research coordinator will obtain their written consent and proceed with the randomization process. |  |
| Inclusion Criteria |  |
| - Ability to speak English and/or Mandarin. - Singaporeans/ Singapore Permanent Residents. - Aged 21 years and above - LACE score >=10 |  |
| Exclusion Criteria  - Subject is a non-resident - Subject has no local home address - Subject is from a long-term care facility during index admission (e.g. nursing homes, inpatient hospices etc) - Subject is unable to participate in telephone survelliance - Subject is discharged before takeover (e.g.discharge planned within 24hrs) - Subject has impaired decision making capacity without surrogate decision maker - Subject is critically ill - Subject or caregiver is mentally unstable - Subject is haemodynamically unstable (e.g. requiring inotropes, blood transfusion etc) - Subject requires acute inpatient respiratory support (eg. Ventilators, Fi O_2_> 50%) - Subject requires acute inpatient dialysis support - Subject pending surgical intervention - Subject pending transfer to other specialist discipline - Primary medical team consultant declined to participate in this research |  |
| **STUDY DESIGN AND PROCEDURES/METHODOLOGY** |  |
| Our study design is an open label, outcome assessor blinded randomised controlled trial.  Randomisation: 840 patients will be randomized in a 1:1 ratio using blocks of 6 into the intervention and control groups. Randomisation sequence will be generated by computer software. Allocation concealment is effected via an off-site telephone service maintained by a hospital administrator.  On every working day, the study team members will receive a list of patients who has been readmitted in the past 90 days. The study team members will then screen through the list for eligibility criteria and their LACE scores. For suitable patients, they will be approached and recruited by the research coordinator at the bedside during their hospitalisation. After consent, randomisation will be carried out. Patients allocated to the intervention group are intervened for 90 days and patients allocated to the control group will receive usual care.  Intervention:  The intervention can be divided into 2 parts. Inpatient and outpatient intervention (Appendix 1). The study team is also divided into the 2 teams. Inpatient and outpatient virtual ward (VW) team. The inpatient team is led by an attending family physician, a junior doctor, a nurse case manager, and a part-time (0.1 Full Time Equivalent) pharmacist while the outpatient VW team comprised of an attending family physician and two nurse case managers. A medical social worker who spends 0.5 full time equivalent time in the team supports both the inpatient and outpatient teams.  Inpatient intervention starts when the patient assigned to the intervention is medically stable to be transferred over to the inpatient clinical team. The inpatient team will continue to optimize the patient’s medical conditions and also systematically identify preventable reasons for readmission. Medications will be reconciled with input from the pharmacist and the appropriate community and social services necessary to support the patient’s transition to home will be activated.  Another core component is patient education and coaching. The nurse case managers will use standardized action plans for chronic diseases (examples include congestive cardiac failure, diabetes mellitus, asthma, chronic obstructive pulmonary disease, chronic kidney disease) for patient education. Finally, an individualized care plan complete with written discharge instructions, patients’ appointments, medication changes and the contact information of the outpatient nurse case manager is to be provided to all patients on discharge. The patient’s care is then handed over to the outpatient virtual ward team at the bedside for a seamless information transfer.  At the home setting, the outpatient nurse case manager will follow up with a telephone call within 72 hours of discharge to assess the patient’s condition and ensure adherence to the prescribed care plans and successful activation of community services. This is followed by a home assessment within one week of discharge. During the home assessment, the nurse case manager will assess the patient’s medical condition and health literacy, competency of the care-giver, availability of nursing and home care equipment, adequacy of social support, safety of the home environment and adherence to medication. Each nurse will be in charge of an average of 30 patients at any time during the program.  A multidisciplinary team meeting will be conducted in the morning of every working day. New patients will be discussed and their care plans reviewed. During the meeting, each nurse case manager is to report on the status of patients under her care. Patients who develop urgent deterioration of their chronic conditions and new medical issues will be recalled to the early review clinic to be reviewed by the family physician. Patients who are doing well in the intervention can be reviewed less frequently at the team meetings. Throughout the intervention period, the nurse case managers are contactable during office hours. Scheduled calls to check in on the patients will be made about once a week. Our intervention will last for 90 days after discharge and patients will be discharged at the end of the intervention period to a primary care provider in the community with continuing specialist input as warranted.  Control Group:  Patients in the control group will receive usual medical care provided by the admitting inpatient team of the hospital. On discharge, patients can be referred to primary care provider, specialists in the outpatient clinic and ambulatory community services as considered necessary by the medical team. As part of standard hospital care, patients will receive an abbreviated standardized patient copy of the hospital discharge summary listing their medical diagnoses and medications. For this study, there will be no contact between patients in the control group and the study team throughout the 3-month interval. A scheduled telephone call will be made at the end of the 3-month study period to verify patient outcomes.  Outcomes:  All outcomes are objectively extracted from the hospital electronic health records and the National Electronic Health Records by an assessor blinded to the treatment allocation.  The primary outcome measure is the 30-day unplanned readmission rate.  Other secondary outcomes included:   1. Unplanned readmission rate to any hospital within 90 days, 180 days of discharge, 2. Emergency department (ED) attendance rate within 30 days, 90 days, 180 days of discharge 3. Probability without readmission or death up to 180 days 4. Index admission and post-discharge mortality rate at 90-day 5. Cumulative length of hospital stay at 90 days 6. Number of outpatient specialist clinic visits at 90-day and 180-day |  |
| **DATA ANALYSIS** |  |
| Data Entry and Storage |  |
| Data entry is made by a trained research co-ordinator into Redcap (Research Electronic Data Capture), a free secure, web-based application designed to support data capture for research studies. Data will be stored in Redcap. Only investigators with access to Redcap can access the data.  **6.2 Data Quality Assurance**  Outcome information will be obtained from the hospital electronic health records and the National Electronic Health Records. Phone follow up will be at 90 days to verify the outcomes information. Information will also be downloaded from routinely used critical care monitors, defibrillators and event records. Public accessible death certificate information will also be reviewed.  Additional steps to ensure data quality includes use of standardized forms, uniform criteria for patient recruitment, internal monitoring of data collection, range checks and verification built into the data entry system and a sequence of logic checking and examination of variables. |  |
| **SAMPLE SIZE AND STATISTICAL METHODS** |  |
| Determination of Sample Size |  |
| We hypothesize that our intervention could reduce readmissions by approximately 25% for patients enrolled in the intervention group. We estimate the baseline readmission rate of eligible patients to be 40% within 30 days of discharge. Based on a power of 80%, level of significance at 5%, and attrition at 10%, a sample size of 420 in each group is required. All statistical analysis will be performed on an intention-to-treat basis. |  |
| **7.2 Statistical and Analytical Plan**  The baseline characteristics will be presented as mean ± SD for continuous variables and frequency counts and percentages for categorical variables. For baseline variables, the 2-sample t-test will be used to compare means and the Pearson chi-square test to compare proportions. A Poisson regression model will be used to obtain relative risks of readmission and ED visits. Kaplan-Meier method will be used to study the survival distribution (based on time to readmission or death) of the intervention and control groups. All tests of significance used 95% level (p<0.05). No subgroup analysis was planned in this study. |  |
| **DIRECT ACCESS TO SOURCE DATA/DOCUMENTS** The investigator(s)/institution(s) will permit study-related monitoring, audits and/or IRB review and regulatory inspection(s), providing direct access to source data/document.   **ETHICAL CONSIDERATIONS** This study will be conducted in accordance with the ethical principles that have their origin in the Declaration of Helsinki and that are consistent with the Singapore Good Clinical Practice and the applicable regulatory requirements.  This final study protocol, including the final version of the Participant Information and Informed Consent Form, must be approved in writing by the Centralised Institutional Review Board (CIRB), prior to enrolment of any participant into the study.  The principle investigator is responsible for informing the CIRB of any amendments to the protocol or other study-related documents, as per local requirement. |  |
| Informed ConsentConsent will be taken by the research co-ordinators after explaining the study protocol, obtaining voluntary agreement of the participant, explaining the number of visits and alternatives and after the participant is clear of his/her study rights. |  |
|  |  |
| Confidentiality of Data and Participant Records |  |
| Research data and participant database will be stored in Redcap (Research Electronic Data Capture), a free secure, web-based application designed to support data capture for research studies. Data will be stored in Redcap. Only investigators with access to Redcap can access the data.  Once data entry is completed, all identifiers will be subsequently removed from the dataset. No further follow up (more than 90 days) is required. No coded research data will be disseminated to any third parties not involved in the research study. All patient identifiers will be removed from the database to protect patient confidentiality. |  |
| **FUNDING** | |
| This is an investigator-initiated study, and supported by a project grant from the Agency of Integrated Care Singapore (AIC/RPD/RD-OIC/ICCS/010/05-2011). | |

Appendix 1: Tasks and Responsibilities of the Inpatient Clinical and Outpatient Virtual Ward Teams

| Functions | Tasks | Responsibility | |
| --- | --- | --- | --- |
|  |  | Inpatient team | Outpatient team |
| 1. Reduce polypharmacy, medication conflicts and error | a. Reconciled medications with pre-admission list | x |  |
|  | b. Identify problem medications (Anticoagulant, anti-platelets, diuretics, insulin & inhalers) | x |  |
|  | c. Review medication use & side effects | x | x |
|  | d. Simplification of medication | x | x |
|  | e. Check on medication adherence within 72 hours after index discharge |  | x |
|  | f. Highlight new/changed/discontinued medication to patient/caregiver & other care providers (Direct communication or discharge summary) | x | x |
| 2. Patient support | a. Initiate ACTION PLANs for high risk diagnosis (Heart failure, Asthma, COPD, renal failure, liver cirrhosis, DM) | x |  |
|  | c. Direct contact information for help given to patient | x |  |
|  | d. Check on ACTION plan adherence within 72 hours after index discharge |  | x |
| 3.Appropriate follow-up | a. Identify primary care provider | x | x |
|  | b. Refer patient to community resources | x | x |
|  | c. Consolidate all patients’ medical appointments | x | x |
|  | d. Arrange review of patients within one week after discharge with memo | x |  |
|  | e. Highlight discharge plan to patient/caregiver & other care providers (Direct communication or discharge summary) | x |  |
|  | f. Home visit within one weeks after discharge |  | x |
|  | g. Monitors patient for deterioration requiring early review |  | x |
| 4. Improve patient & caregiver psycho-social status | a. Screen for depression in patient | x | x |
|  | b. Screen for dementia in patient | x | x |
|  | c. Screen for caregiver stress | x | x |
| 5. Practice of preventive medicine | a. Review reasons for readmission | x |  |
|  | b. Fall risk assessment & prevention | x | x |
|  | d. Recommend appropriate lifestyle modification |  | x |
|  | e. Recommend appropriate vaccination | x | x |
| 6. Palliative care | a. Assess need for advance care planning | x | x |
